# A Rare Case of Central Nervous System Vasculitis in a Patient with Perinuclear Antineutrophil Cytoplasmic Antibodies-associated Interstitial Lung Disease

**DOI:** 10.7759/cureus.7144

**Published:** 2020-02-29

**Authors:** Ammar Haikal, Tanawat Attachaipanich, Kyle Myers, Paul Schmidt, Arsany Anis

**Affiliations:** 1 Internal Medicine, University of Kansas Medical Center, Kansas City, USA; 2 Internal Medicine, Faculty of Medicine Siriraj Hospital, Mahidol University, Bangkok, THA; 3 Internal Medicine, Saint Michael's Medical Center, Newark, USA

**Keywords:** anca-associated cns vasculitis, leptomeningeal enhancement, chronic hypertrophic pachymeningitis, interstitial lung disease, rituximab

## Abstract

Antineutrophil cytoplasmic antibody (ANCA)-associated vasculitis (AAV) is a systemic necrotizing inflammation of the small vessels. Central nervous system (CNS) ANCA-associated vasculitis is a rare manifestation of AAV. Three mechanisms of AAV affecting the CNS have been reported which include contiguous granulomatous invasion from nasal and paranasal sinuses, remote granulomatous lesions, and vasculitis of small vessels. Chronic hypertrophic pachymeningitis (CHP) is the meningeal-site involvement in AAV caused by granulomatous inflammation in the dura mater. We present a case of pachymeningitis manifested with slowly progressive cognitive dysfunction, leptomeningeal enhancement on MRI, and necrotic vessels with surrounding inflammation on biopsy. This case represents a rare development of subsequent CNS AAV in a patient with ANCA-associated interstitial lung disease treated with rituximab with a resolution of leptomeningeal enhancement on a follow-up magnetic resonance imaging (MRI).

## Introduction

Pachymeningitis is fibrous inflammatory changes involving the central nervous system (CNS) dura matter. Various etiologies for pachymeningitis were identified including infectious diseases such as tuberculosis, syphilis, cryptococcal infection, and Lyme disease; autoimmune or inflammatory diseases such as granulomatosis with polyangiitis (GPA), sarcoidosis, and immunoglobulin G4 (IgG4)-related disease; and malignancies, in particular, lymphoma [1]. Clinical presentation depends on the location of the CNS involvement. Early diagnosis of pachymeningitis is critical in preventing neurological damage.

Chronic hypertrophic pachymeningitis (CHP) is the meningeal involvement in CNS antineutrophil cytoplasmic antibody (ANCA)-associated vasculitis (AAV). We present a case highlighting the rare development of AAV-associated pachymeningitis in a patient with prior ANCA-associated interstitial lung disease, treated with rituximab with a resolution of leptomeningeal enhancement on follow-up magnetic resonance imaging (MRI).

## Case presentation

A 76-year-old Hispanic female presented with progressive forgetfulness, depression and personality changes over a few weeks. She was subsequently admitted to the hospital after the MRI brain showed leptomeningeal enhancement and multifocal white matter fluid-attenuated inversion recovery (FLAIR) hyperintensities. She also has interstitial lung disease (usual interstitial pneumonitis (UIP)) with positive perinuclear ANCA (p-ANCA) treated with rituximab three years prior to presentation. Serial pulmonary function tests (PFTs) and a follow-up chest computed tomography (CT) were stable. Her physical exam was notable for known basilar dry rales in both lungs, newly noticed low cognitive testing and flat affect.

Laboratory evaluation revealed hemoglobin 11.1 (normal range: 12.0-15.0 g/dL), white blood cell count (WBC) 10.7 (normal range: 3.8-10.8 K/uL), platelet 365,000 (normal range: 150-400 K/uL), erythrocyte sedimentation rate 96 mm/hr (normal range: 0-29 mm/hr) and C-reactive protein 3.05 mg/dL (normal range: <3 mg/dL). Urinalysis showed no proteinuria or hematuria. Antinuclear antibodies by enzyme-linked immunosorbent assay (ELISA) was positive with a titer of 1:160, homogeneous pattern (normal range: < 1:40). Anti-double-stranded deoxyribonucleic acid (anti-dsDNA) antibody, anti-Sjögren's syndrome-related antigen A/antigen B (SSA/SSB) antibody, anti-ribonucleoprotein (RNP), and anti-Smith antibodies were negative. Anti-cyclic citrullinated peptide antibodies were negative, but rheumatoid factor (RF) was 115 (normal range: <24 IU/ml). Repeat ANCA by immunofluorescence and ELISA showed positive p-ANCA/myeloperoxidase antibody (more than eight), negative cytoplasmic-ANCA (c-ANCA) and normal serum complement levels. Lumbar puncture revealed WBC 15 cells/µL with 58% lymphocytes (normal range: < 5 cells/µL with 60%-70% lymphocytes), protein 141 mg/dL (normal range: 15-45 mg/dL) and glucose 57 mg/dL (normal range: 40-75 mg/dL). Serology for Epstein-Barr virus, herpes simplex virus, enteroviruses, cytomegalovirus, and varicella zoster virus, bacterial and fungal cultures, were negative. Magnetic resonance angiography (MRA) showed a patent intracranial vasculature without evidence of luminal irregularities. 

**Figure 1:**
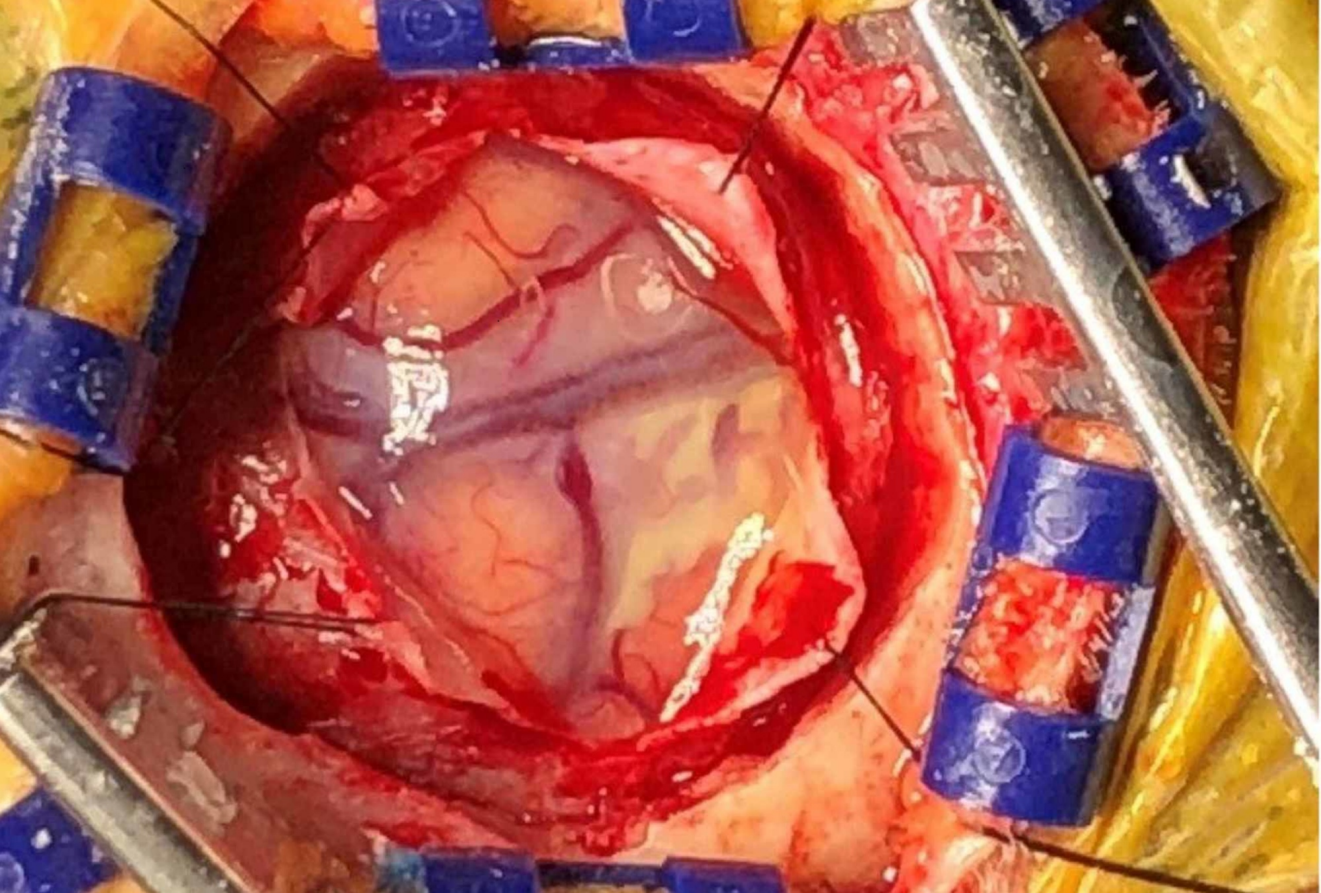
Theleptomeninges showed some white-yellow thickening grossly

**Figure 2:**
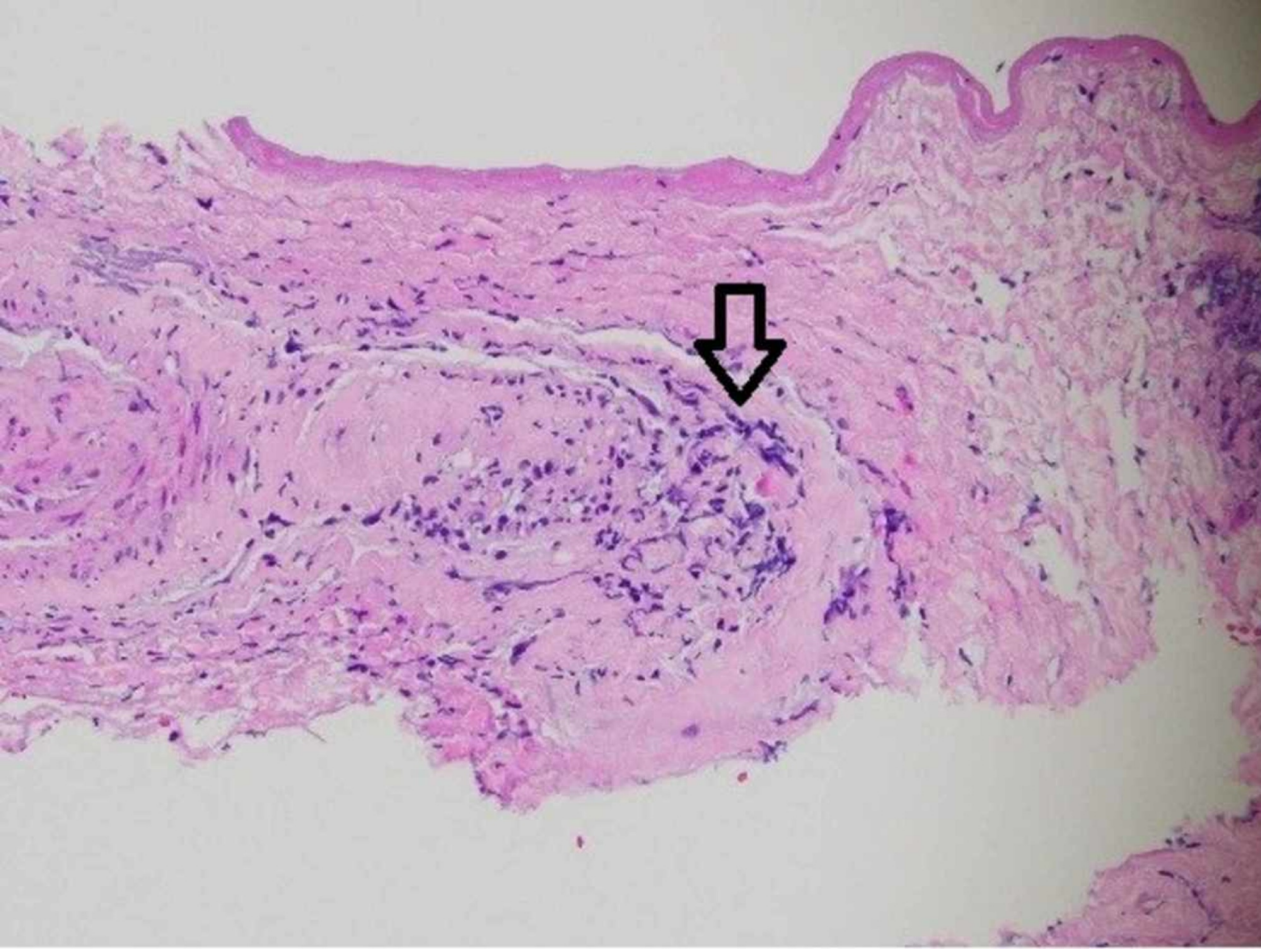
High power with a vessel and adjacent inflammatory cells

**Figure 3:**
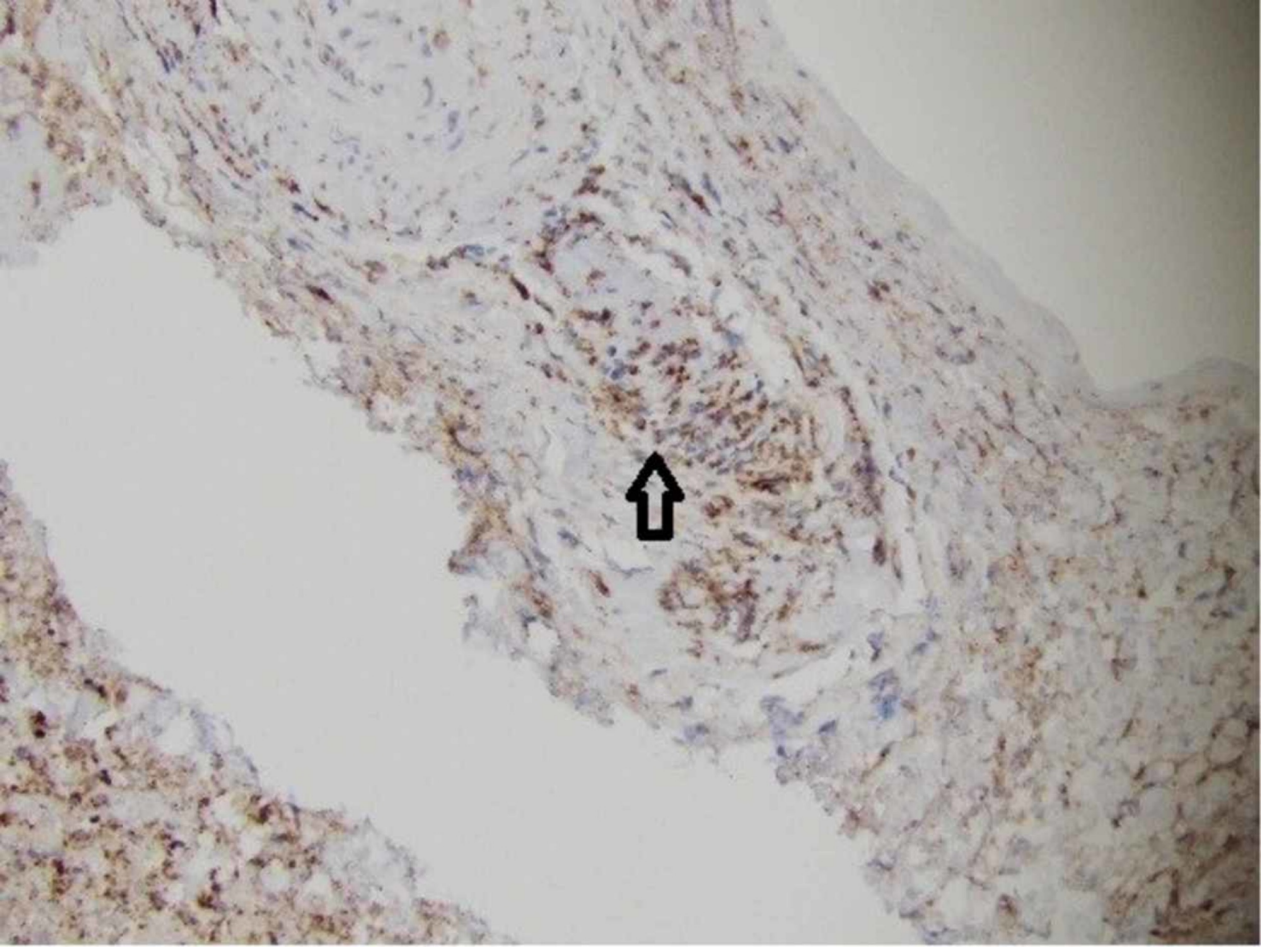
+Cluster of differentiation (CD) 68 macrophages

**Figure 4:**
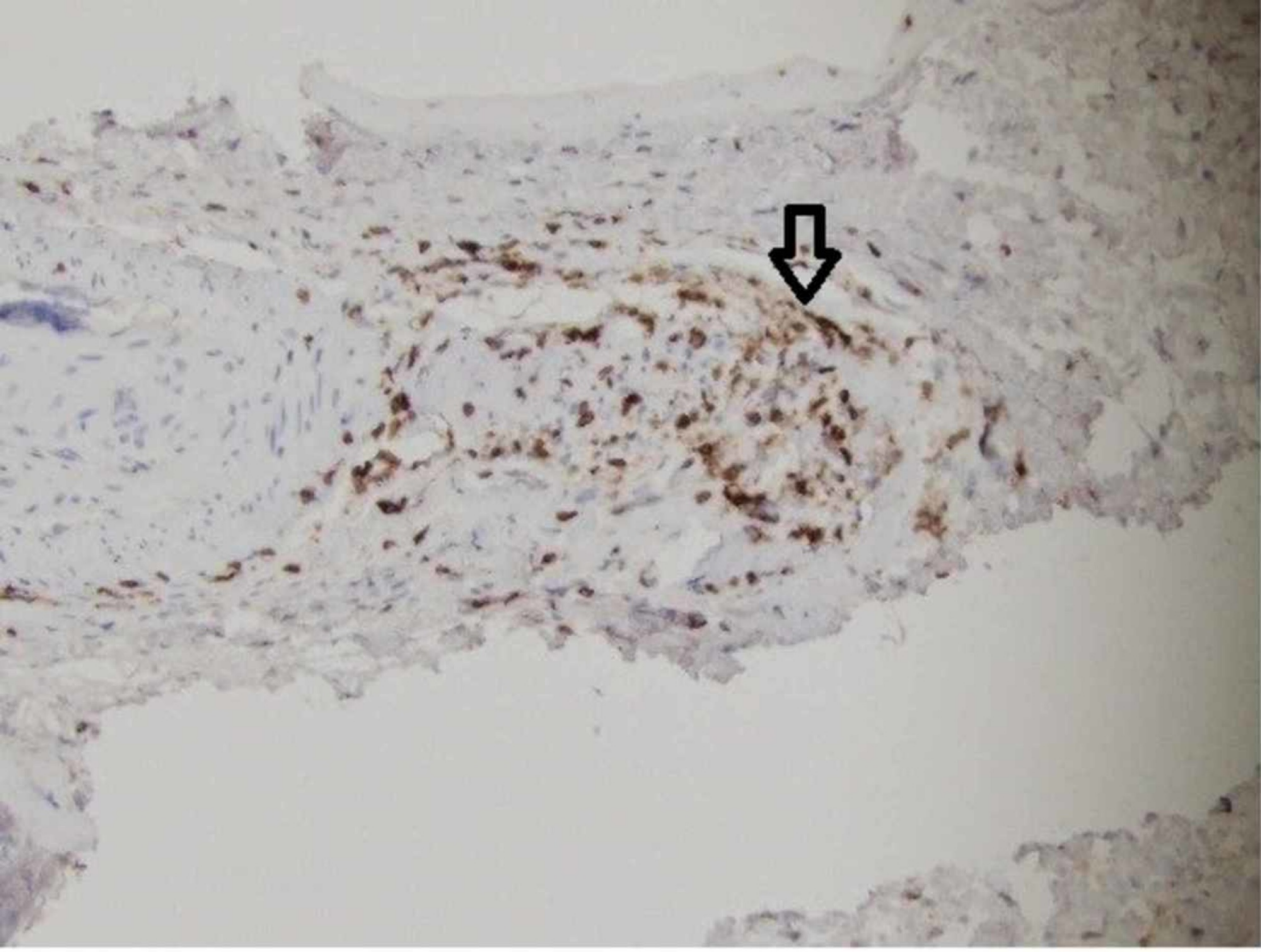
+Cluster of differentiation (CD) 3 positive T-cells

A leptomeningeal biopsy was performed, with the surgeon commenting on grossly abnormal white-yellow thickened leptomeningeal tissue (Figure 1). The biopsy showed a necrotic vessel surrounded with macrophages and T-cells, with necrosis within the vascular lumen and within the vascular wall (Figures 2-4). There was no evidence of lymphoma (CD20 negative) and no organisms identified. Beta-amyloid stain was negative. Based on the negative infectious and malignant workups, it was felt the leptomeningeal enhancement was related to p-ANCA-associated vasculitis. The patient was treated with intravenous methylprednisolone 1000 mg/day for three days, then transitioned to prednisone 60 mg daily with scheduled appointment for follow-up in our rheumatology clinic. Her family noticed a dramatic improvement in her alertness. Rituximab (375 mg/m2 once weekly for four weeks) induction therapy was given after discharge. MRI brain three months later showed resolution of leptomeningeal enhancement. Her cognition improved at her most recent cognitive evaluation. She currently lives in an assisted living facility.

## Discussion

AAV is a systemic vasculitis characterized by necrotizing inflammation of small-sized blood vessels with few or no immune deposits [2]. Major variants include GPA, microscopic polyangiitis (MPA), eosinophilic granulomatosis with polyangiitis (EGPA) and a single-organ AAV [2]. Neurologic involvement in AAV has been reported in approximately 34%-54% of cases with the majority involving cranial neuropathies [3-4].

There are three pathogenic mechanisms described: first, granulomatous contiguous invasion from nasal and paranasal sinuses causing cranial nerve palsies, meningeal involvement and pituitary gland hormonal dysfunction; second, remote granulomatous lesions, less common than contiguous invasion, causing intracerebral granuloma and cranial nerve involvement; and third, vasculitis of small blood vessels causing ischemic and/or hemorrhagic complications [5].

Our patient had a leptomeningeal enhancement which is a representation of CHP. CHP is the meningeal-site involvement in AAV and caused by granulomatous inflammation at the dura mater [6]. The prevalence of CHP in AAV is 17.9%, with most cases associated with GPA and a few with MPA, and is seen at initial presentation in 42.9% of cases [6-7]. The intracranial dura mater is more commonly affected than the spinal cord. Meningeal involvement causes various clinical presentations including headache, cranial neuropathies, cerebellar ataxia, seizure, myelopathy and various ocular manifestation [7-8]. The presence of CHP is associated with concomitant paranasal sinus involvement but not with renal involvement, which supports the pathogenesis of CHP as granulomatous inflammation [7-8]. ANCA serology may correlate with the severity of the disease. Anti-proteinase-3 positive-CHP is more likely to have leptomeningeal and parenchymal involvement, is associated with more severe neurological sequelae, and tends to show renal and pulmonary systemic involvement. Conversely, anti-myeloperoxidase positive-CHP is more likely to have CNS-limited disease with lower relapse rates [8]. The cerebrospinal fluid analysis in CHP may show elevated protein with mild pleocytosis while MRI shows thickening of dura mater with gadolinium enhancement [5-8]. Most histopathological studies reveal a thickening of the dura, with 50% showing necrotizing granulomatous inflammation and vasculitis [7-8].

Our patient presented with interstitial lung disease three years after diagnosis with cognitive impairment and leptomeningeal enhancement on MRI without another active organ involvement. She was diagnosed with ANCA-associated CNS vasculitis. There is evidence that a significant number of patients with interstitial lung disease and positive ANCA - especially anti-MPO ANCA - will go on to develop microscopic polyangiitis [9]. Interestingly, a UIP pattern was the most common form of interstitial lung disease in this cohort, as found in our patient. There are few studies about cognitive impairment in AAV. About 30% of non-demented AAV patients also have subclinical cognitive impairment [10]. However, disease duration and severity are not predictors of subclinical dementia in AAV [10]. Our patient was treated with rituximab with resolution of prior MRI leptomeningeal enhancement. Rituximab has been shown to be non-inferior to cyclophosphamide for induction of remission and may be superior for relapsing disease [11]. It remains challenging to assess cognitive response and attribute deficits with certainty to AAV versus other etiologies.

## Conclusions

CHP is a rare disease resulting from meningeal involvement in CNS AAV. ANCA serology, MRI findings, and leptomeningeal biopsy help in making the diagnosis. Early detection of pachymeningitis is crucial in preventing permanent neurological damage. CNS AAV is typically treated similarly to AAV with other systemic involvement, with remission-induction therapy consisting of high-dose corticosteroids and rituximab. More studies are needed to assess the most effective and safest immunosuppressive therapy for ANCA-associated CNS vasculitis.
